# H_2_O_2_ ‐Producing Electrochemical Bandages Are Active Using Off‐the‐Shelf Hydrogels

**DOI:** 10.1111/wrr.70092

**Published:** 2025-09-15

**Authors:** Eda Dagsuyu, Paige Kies, Robin Patel, Haluk Beyenal

**Affiliations:** ^1^ School of Chemical Engineering and Bioengineering, Voiland College of Engineering and Architecture Washington State University Pullman Washington USA; ^2^ Department of Chemistry, Faculty of Engineering Istanbul University‐Cerrahpaşa Istanbul Turkey; ^3^ Division of Clinical Microbiology Mayo Clinic Rochester Minnesota USA; ^4^ Division of Public Health, Infectious Diseases, and Occupational Medicine, Department of Medicine Mayo Clinic Rochester Minnesota USA

**Keywords:** biocide, biofilm, e‐bandage, hydrogel, hydrogen peroxide, wound healing

## Abstract

An electrochemical bandage (e‐bandage) that generates hydrogen peroxide (H_2_O_2_) through a combination of working, counter and reference electrodes used with an electrolyte‐providing hydrogel is being developed for wound infection management. e‐Bandage biocidal activity was previously demonstrated using Xanthan gum hydrogel. For clinical use, clinically used hydrogels would be ideal, but their use with the described e‐bandage has not been shown. The goal of this work was to evaluate the biocidal activity of off‐the‐shelf, clinically used hydrogels when used with a 1.77 cm^2^ H_2_O_2_‐producing e‐bandage. e‐Bandage electrochemical parameters and activity against methicillin‐resistant *Staphylococcus aureus* IDRL‐6169 and *Acinetobacter baumannii* ATCC‐17978 biofilms were assessed with six off‐the‐shelf hydrogels. Variations in hydrogel composition affected electrochemical parameters, which was associated with differences in biocidal activity. Results of this study inform the selection of off‐the‐shelf hydrogels for use with H_2_O_2_‐producing e‐bandages.

## 1 Introduction

Chronic wounds are wounds that do not complete a normal healing process; they typically require long‐term care [1]. These wounds may result from pressure injuries, diabetes and venous or arterial insufficiency [2]. Individuals with diabetes and elevated body mass index are at increased risk of developing chronic wounds [3]. Approximately 2.5% of the United States population is affected by chronic wounds at any given time, and the global wound care market is projected to grow by 4.61% from 2023 to 2030 [4]. The cost of treating diabetic foot wounds and venous ulcers is approximately $10–$15 billion annually in the United States [5, 6]. Those affected may experience pain, emotional and physical distress, reduced mobility, and/or social isolation [7].

When skin injury occurs, bacteria from skin microbiota and/or contaminating material colonize the wound [8]. While the immune response typically keeps colonizing bacteria in check, if wound healing is delayed, and/or pathogenic microbes are present, clinically significant infection may occur [9]. Pathogens that cause wound infections include *Staphylococcus aureus* , *Pseudomonas aeruginosa* , *Acinetobacter baumannii* and *Candida* species, amongst others [4]. These organisms form biofilms on necrotic tissues [10]. Methicillin‐resistant *S. aureus* (MRSA) is a particular challenge [11]; similarly, antibiotic‐resistant *A. baumannii* can flourish in hospital environments and causes 2% of intensive care unit‐acquired skin/soft tissue infections [12].

Hydrogen peroxide (H_2_O_2_) is a reactive oxygen species (ROS) that incites DNA damage and membrane lipid peroxidation, alters membrane potential and oxidizes thiol‐containing proteins, which can lead to cellular death [13]. H_2_O_2_ is a broad‐spectrum antiseptic, targeting bacteria and fungi. Neutrophils and macrophages generate and utilize H_2_O_2_ to clear bacteria that colonize wound beds; this biocide is also a signaling molecule involved in the wound healing process [13, 14]. For this reason, there is interest in leveraging H_2_O_2_ to treat and prevent wound infections, including those caused by antibiotic‐resistant bacteria.

To this end, a H_2_O_2_‐producing electrochemical bandage (e‐bandage) is being developed for wound infection prevention and treatment, as well as wound healing [15, 16, 17, 18, 19, 20]. The e‐bandage is an electrochemical system with working, counter, and reference electrodes designed to continuously generate low concentrations of H_2_O_2_ via partial reduction of oxygen when operated at −0.6 V_Ag/AgCl_ (O_2_ + 2H^+^ + 2e^−^ ⟺ H_2_O_2_ E^0^ = +0.085 V_Ag/AgCl_). Electrodes are soaked in phosphate‐buffered saline (PBS) and separated by cotton fabric layers and a hydrogel. A hydrogel which contains water and ions (e.g., Na^+^, Cl^−^) provides an electrolyte source [21, 22, 23].

Beyond being an integral part of the electrochemical system, for wound therapy, hydrogels provide biocompatibility and a connection between the e‐bandage and the wound [24, 25]. Unlike traditional dry electrode materials, the water‐ and ion‐rich composition of hydrogels can resembles biological tissues, enabling mechanical and ionic compatibility, conductivity, flexibility and even responsiveness to external stimuli [26, 27, 28]. Many off‐the‐shelf hydrogels are used for wound healing and infection prevention. Hydrogels may support wound healing by creating a moist and cooling environment, due to their high water content and customizable properties based on polymer selection and crosslinking methods [29]. Off‐the‐shelf products include alkene polymer‐based, cross‐linked hydrophilic and cellulose‐based hydrogels used for drug delivery and tissue engineering, and those made of polyethylene glycol/polyester block copolymers with stimuli‐responsive functions used for healing properties, controlled drug release, and/or sensing [30, 31, 32]. While some chemicals used in hydrogels may have side effects (e.g., skin corrosion/irritation, skin sensitization) [33], many are considered safe and are approved by the United States Food and Drug Administration (FDA) for use on humans—for example, no adverse effects have been reported with the use of 3M hydrogel [34]. The H_2_O_2_‐producing e‐bandage is being developed for human use and could theoretically be deployed with clinically used hydrogels. However, clinically used hydrogels have not been tested in electrochemical systems, and their suitability for such an application is not defined.

The goal of this work was to determine whether off‐the‐shelf, FDA‐approved clinical hydrogels can be integrated with H_2_O_2_‐producing e‐bandages. This study evaluated the impact of off‐the‐shelf hydrogels on e‐bandage biocidal activity against MRSA IDRL‐6169 and *A. baumannii* ATCC 17978 biofilms. A previously described 1.77 cm^2^ e‐bandage [17, 18, 20, 35] was used with six off‐the‐shelf hydrogels (3M, Duoderm, Prontosan, Purilon, Skintegrity and Solosite) to which 0.9% NaCl was added to operate the e‐bandage electrochemically, in comparison to Xanthan gum hydrogel (used in prior e‐bandage evaluations [17, 18, 20, 35]) as a control. One of the off‐the‐shelf hydrogels—3M hydrogel—was also tested with added water content to assess the effect of this modification on biocidal activity, as higher water content is expected to enhance ion mobility within the hydrogel while maintaining a moist environment conducive to wound healing. Since chemicals in off‐the‐shelf hydrogels might dissolve e‐bandage carbon fabric binder matrices, two counter electrode (CE) materials (Elat and Panex) were tested. Verification of e‐bandage operation using FDA‐approved clinical hydrogels and two different electrode materials is expected to advance the technology towards clinical application in humans.

## 2 Experimental

### 2.1 Electrochemical Bandage (e‐Bandage)

**FIGURE 1:**
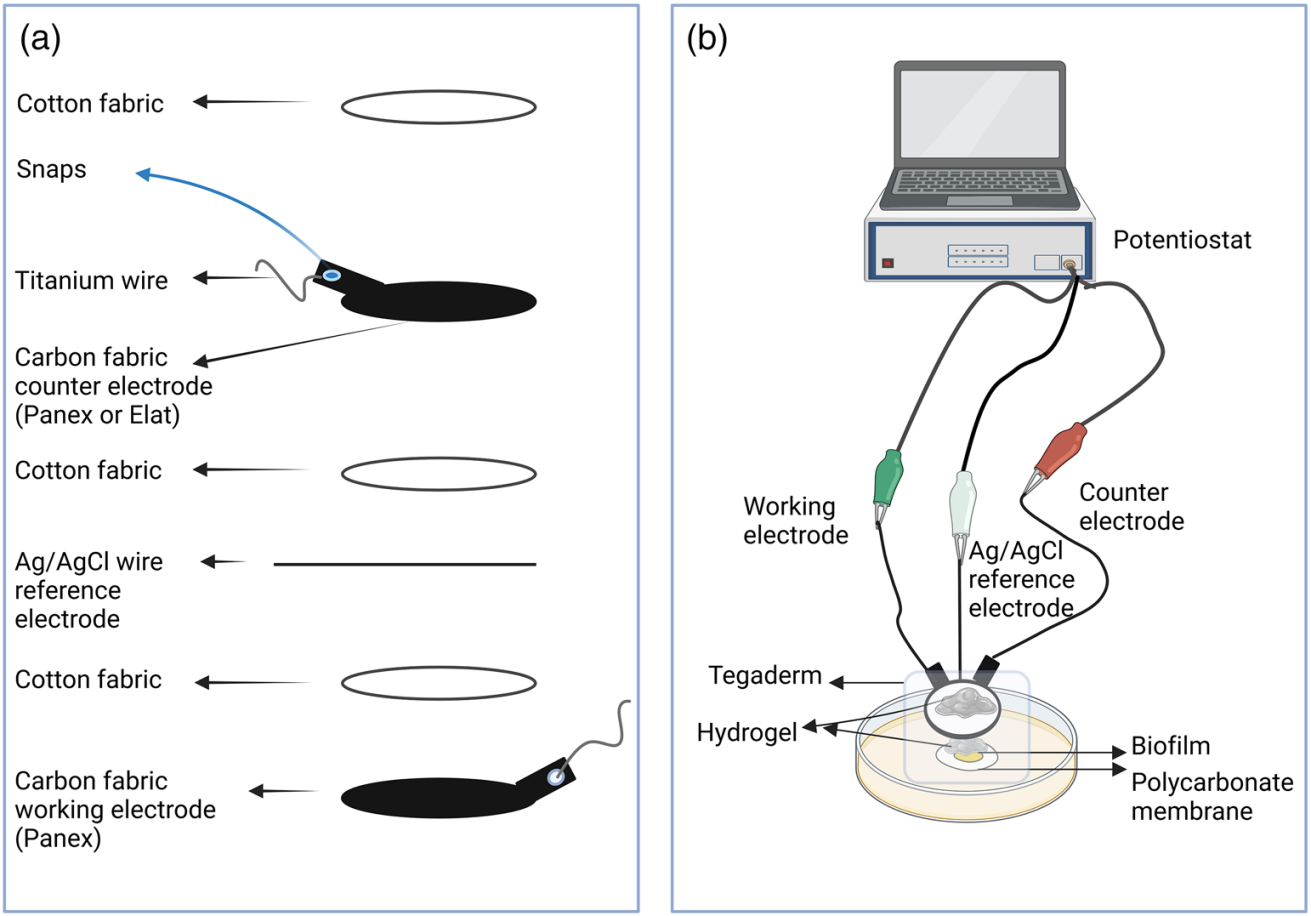
(a) Schematic representation of e‐bandage layers; and (b) the in vitro experimental setup comprises e‐bandages interfaced with a potentiostat, applied to laboratory‐cultured biofilms on a tryptic soy agar plate (created with BioRender.com).

Construction, application, and details of e‐bandages are covered in previous publications [17, 19, 20, 35]. The e‐bandage is an electrochemical system composed of three electrodes: two layers of carbon fabric serving as the working electrode (WE, 1.77 cm^2^, Panex 30 PW‐06, Zoltek Companies Inc.) and as the CE (Panex or Elat‐Product code: 1591001, Fuel cell store, USA) and a silver/silver chloride (Ag/AgCl) wire functioning as a quasi‐reference electrode (QRE). The electrodes are separated by three layers of cotton fabric (2.25 cm^2^ each) and held together using silicon adhesive. Electrical connectivity is established via 30 AWG titanium wires (TEMco, Amazon.com, catalog no. RW0517) pressed with nylon sew‐on snaps (Dritz, Spartanburg, SC, item no. 85), which link the carbon fabric electrodes to a potentiostat cable (Interface 1010T/Interface 1000, Gamry) with a multiplexer (IMX8/ECM8, Gamry) (Figure 1a). The WE and CE are covered with a hydrogel to ensure electrochemical connectivity. Before experiments, e‐bandages are soaked in PBS for approximately 15 min, with hydrogel (100 μL) added between the layers to ensure all components are adequately covered.

### 2.2 Preparing of Hydrogels

3M (3M, US), Duoderm (Convatec, UK), Prontosan (Bbraun Medical Inc., Germany), Purilon (Coloplast, Denmark), Skintegrity (Medline Industries, US) and Solosite (Smith & Nephew Medical Limited, US) hydrogels were studied. NaCl (0.76 g) was added to each of these hydrogels per 100 g. Contents of the purchased hydrogels are shown in Table 1.

**TABLE 1:** Hydrogel ingredients.

Brand	Glycerol	Carboxtmethlycellose sodium	Allantoin	Benzyl alcohol	Methyl paraben	Propylparaben	Hydroxyethyl cellulose	Betaine	Polyaminopropyl biguanide	Dextran	Sodium benzoate	Pectin	Calcium alginate	Propylene glycol	Guar gum	Sodium tetraborate
3M hydrogel [34]														+	+	+
Duoderm Gel [36]		+										+				
Prontosan wound gel X [37]	+						+	+	+							
Purilon gel [38]		+											+			
Skintegrity hydrogel [39]	+		+				+			+	+					
SoloSite wound gel [40]	+	+	+	+	+	+										

PBS solution was prepared by dissolving Na_2_HPO_4_ (0.01 M), KH_2_PO_4_ (0.0018 M), NaCl (0.137 M) and KCl (0.0027 M) into 18 MΩ cm DI water (1 L). Xanthan gum (Namaste Foods, Amazon.com, UPC: 301155217160) hydrogel was made from 1× PBS mixed with Xanthan gum (1.8% [wt/vol]). 3M derived hydrogels were prepared as described in Table 2. All hydrogels were autoclaved at 121°C for 15 min under liquid cycle conditions.

**TABLE 2:** Composition of 3M derived hydrogel ingredients.

Ingredient	% By weight manufacturer information	Composition to trial for 100 g		
		3M_Max_	3M_Med_	3M_Min_
Water	70–95	93.15 g	84.15 g	74.15 g
Propylene glycol	5–20	5.00 g	12.50 g	20.00 g
Guar gum	1–5	1.00 g	2.50 g	5.00 g
Sodium tetraborate	< 0.1	0.09 g	0.09 g	0.09 g
NaCl		0.76 g	0.76 g	0.76 g

### 2.3 In vitro Agar Membrane Biofilm Model

An agar wound biofilm model that mimics wound beds was used [16, 35, 41]. Antibiofilm activity of H_2_O_2_‐producing e‐bandages against MRSA IDRL‐6169 and *A. baumannii* ATCC 17978, representing species routinely associated with wound biofilm infections, was assessed. For the in vitro agar membrane biofilm model, each isolate was streaked for isolation from freezer stocks (−80°C) onto tryptic soy agar (TSA) plates and incubated at 37°C for 24 h. Colonies were then used to inoculate 2 mL tryptic soy broths (TSBs), which were incubated at 37°C at 150 rpm until reaching 0.5 McFarland turbidity (approximately 50 min). Then, 2.5 μL of the 0.5 McFarland culture was spotted on the center of UV‐sterilized 13 mm polycarbonate membranes (Whatman Nuclepore polycarbonate hydrophilic membranes, Cytiva no. 10417001) atop TSA plate. Inoculated membranes were incubated for 24 h at 37°C to establish a biofilm.

### 2.4 e‐Bandage Treatment

Twenty‐four hour agar biofilms were transferred to a fresh TSA plate. Hydrogel (100 μL) was placed between the cotton fabric layers of the e‐bandage, where the QRE was situated, using a syringe needle. Hydrogel (100 μL) was placed on top of the biofilm, and an e‐bandage was placed on top of the hydrogel, with the WE side in contact with the biofilm side for non‐polarized and polarized groups. Then, another 100 μL of hydrogel was placed on top of the e‐bandage. A sterile Tegaderm transparent film (3M, reference no. 1622W) was used to cover the e‐bandage. e‐Bandage wires were taped to the side of the TSA plate. Then, the lid was placed on the TSA plate, and the plates were wrapped with parafilm. Each e‐bandage was connected to a potentiostat (Interface 1010T/Interface 1000, Gamry) with a multiplexer (IMX8/ECM8, Gamry). The WE was polarized to −0.6 VAg/AgCl to generate H_2_O_2_ on the WE surface (Figure 1b). Nonpolarized (control; no production of oxidizing agents) conditions used the same setup without polarization. The effects of different hydrogels against MRSA IDRL‐6169 or A. baumannii ATCC 17978 agar membrane biofilms were examined with electrochemically generated H_2_O_2_ utilizing the e‐bandages. Biofilms were treated for 24 h (polarized group).

### 2.5 Biofilm Quantification

e‐Bandages were removed from TSA plates and placed in sterile Petri dishes. Biofilms were scraped from the WE surface into PBS (5 mL). The PBS solution and the membranes were transferred to 15‐mL centrifuge tubes, vortexed for 2 min and sonicated for 10 min at 50 Hz. Following this, cells were pelleted by centrifugation at 2910 relative centrifugal force for 10 min and resuspended in 1 mL PBS. The final 1 mL resuspension was serially diluted (10‐fold), and 10 μL of each dilution was spotted on TSA and incubated at 37°C overnight. Colony‐forming units (CFU) were counted after 24 h [42].

### 2.6 Statistical Analysis

Data were displayed as individual data points for at least four biological replicates (i.e., results of experiments performed on different days) with standard deviations. Comparisons between groups were carried out using the Wilcoxon rank sum test. Non‐parametric tests were selected. All tests were two‐tailed, with statistical significance considered for *p* values below 0.05. Statistical analysis and figures were generated using GraphPad Prism (GraphPad Software 10.4.1).

## 3 Results

To evaluate compatibility of off‐the‐shelf hydrogels with e‐bandages, custom‐prepared 3M‐derived and Xanthan gum hydrogels were tested with H_2_O_2_‐producing e‐bandages and hydrogels for their ability to reduce MRSA IDRL‐6169 and *A. baumannii* ATCC 17978 burden in vitro. Additionally, to assess compatibility of the e‐bandages with different CE materials (Elat and Panex carbon fabric) were tested with all hydrogels.

### 3.1 Activity of Off‐the‐Shelf Hydrogels Against MRSA IDRL‐6169 Biofilms

**FIGURE 2:**
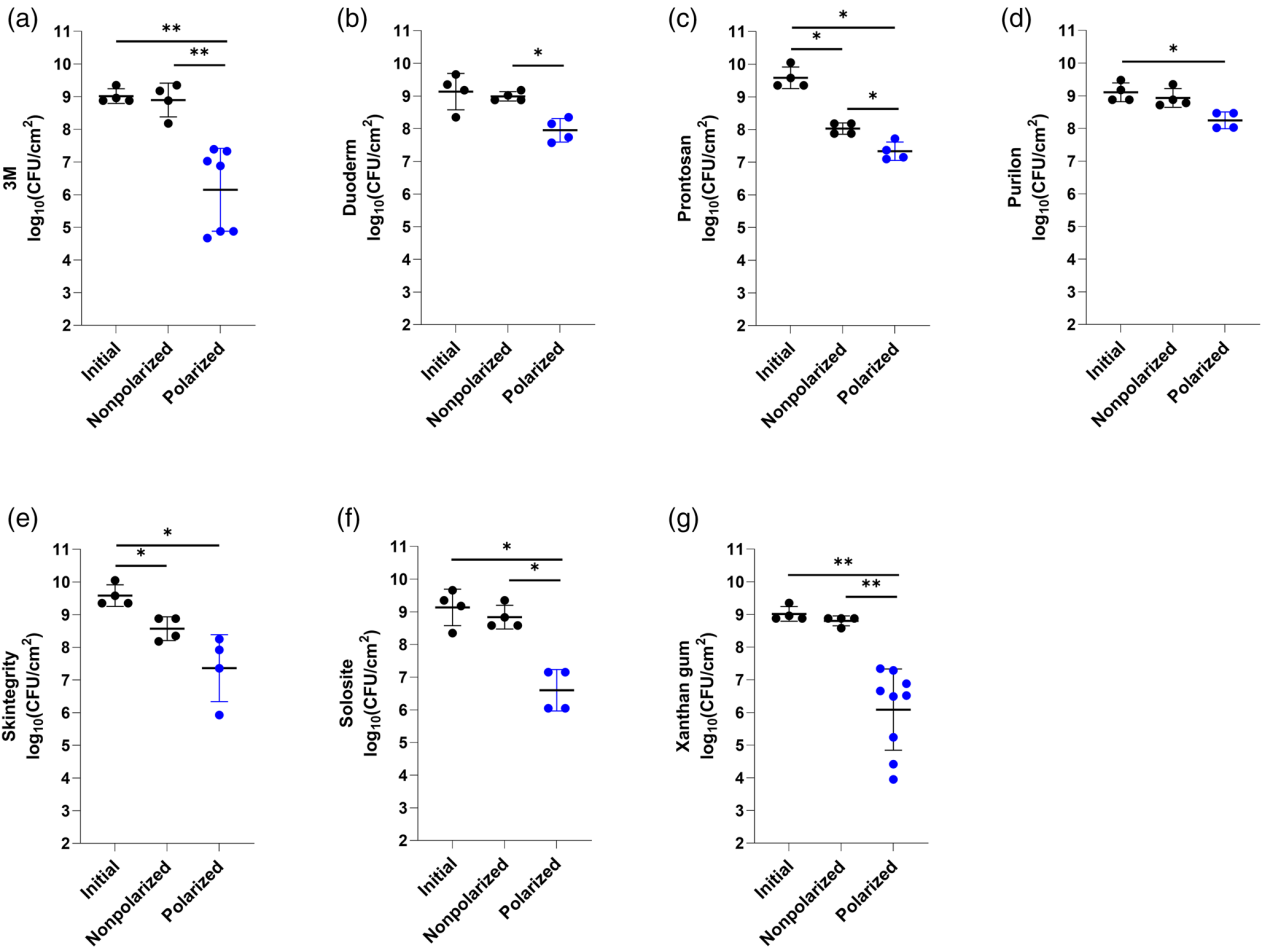
Results of testing of Panex counter and working electrode‐constructed e‐bandages against methicillin‐resistant *Staphylococcus aureus* IDRL‐6169 with (a) 3M, (b) Duoderm, (c) Prontosan, (d) Purilon, (e) Skintegrity, (f) Solosite and (g) Xanthan gum hydrogels. Polarized groups (shown in blue) had e‐bandages polarized for 24 h. Nonpolarized groups had e‐bandages applied but not polarized for 24 h. ‘Initial’ designates initial bacterial quantities (i.e., at time zero). Data points represent individual biological replicates (circles) and means (horizontal lines) with standard deviations of at least four independent biological replicates with statistically significant comparisons (*p < 0.05, **p < 0.01, two‐sided Wilcoxon rank‐sum test) shown.

**FIGURE 3:**
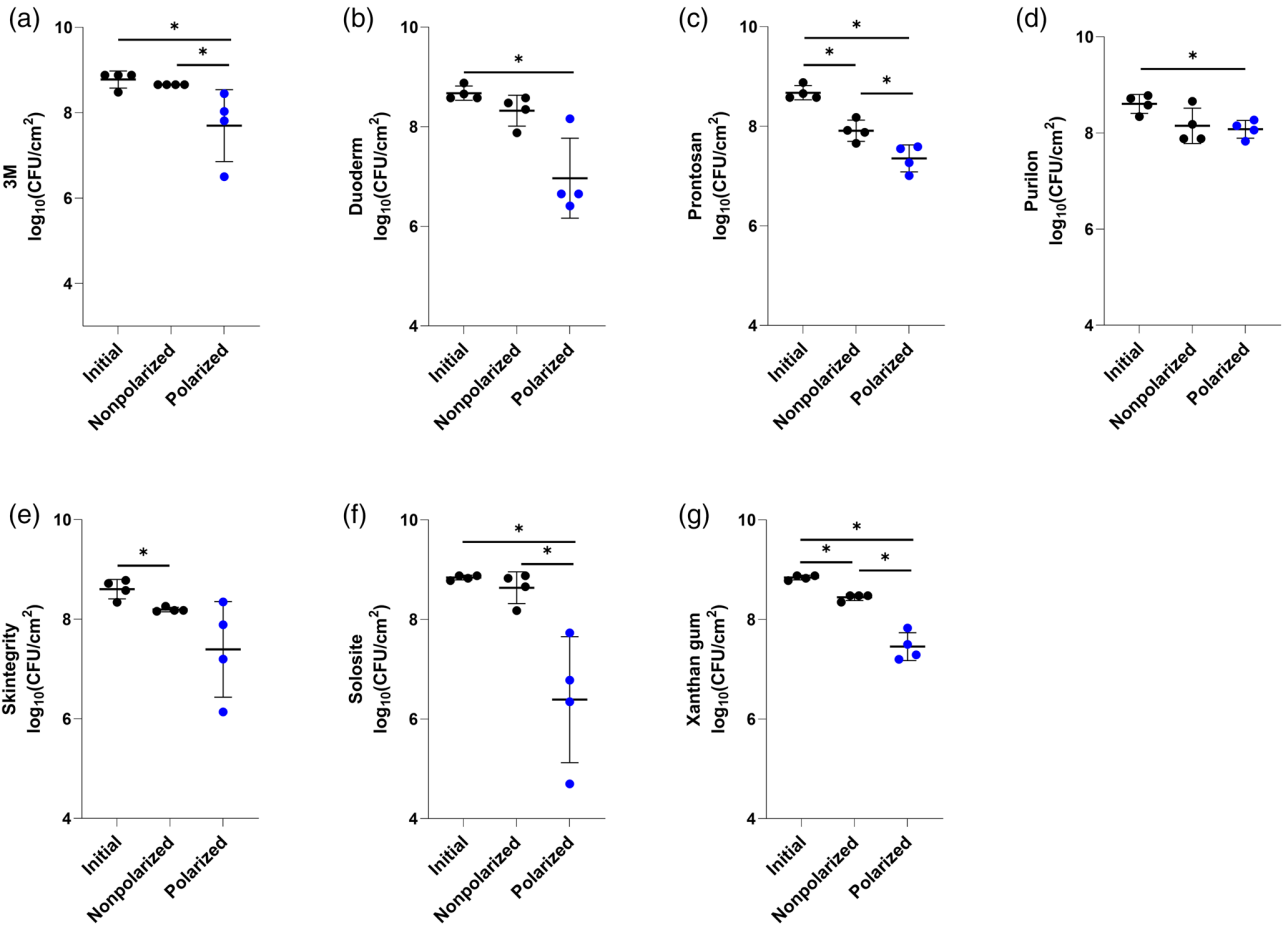
Results of testing of Elat counter electrode‐ and Panex working electrode‐constructed e‐bandages against methicillin‐resistant *Staphylococcus aureus* IDRL‐6169 with (a) 3M, (b) Duoderm, (c) Prontosan, (d) Purilon, (e) Skintegrity, (f) Solosite and (g) Xanthan gum hydrogels. Polarized groups (shown in blue) had e‐bandages polarized for 24 h. Nonpolarized groups had e‐bandages applied but not polarized for 24 h. ‘Initial’ designates initial bacterial quantities (i.e., at time zero). Data points represent individual biological replicates (circles) and means (horizontal lines) with standard deviations of at least four independent biological replicates with statistically significant comparisons (*p < 0.05, two‐sided Wilcoxon rank‐sum test) shown.

Figures 2 and 3 show e‐bandage activities with six off‐the‐shelf hydrogels and Xanthan gum against MRSA IDRL‐6169 biofilms when using Panex and Elat carbon fabric as the CE, respectively.

Since previous studies operated the e‐bandage with Xanthan gum, it was used as a control. Polarized Panex CE‐constructed e‐bandages using Xanthan gum hydrogel showed a significant decrease in MRSA IDRL‐6169 burden compared to nonpolarized e‐bandages (*p* < 0.01, Figure 2g); there was no reduction between the initial and nonpolarized e‐bandage groups, suggesting that Xanthan gum alone does not impact MRSA IDRL‐6169 quantities. Xanthan gum (control hydrogel), with the polarized e‐bandages, resulted in a similar reduction in bacterial load of MRSA IDRL‐6169 (~3–4 log_10_ [CFU/cm^2^]) as in our prior study [16]. Polarized Panex CE‐constructed e‐bandages using 3M, Duoderm and Solosite hydrogels showed similar effects to Xanthan gum (Figure 2a,b,f, respectively). Prontosan and Skintegrity hydrogels alone with nonpolarized e‐bandages resulted in decreases in MRSA IDRL‐6169 compared to initial quantities, implying that these hydrogels may have independent biocidal activity (*p* < 0.05, Figure 2c,e). Interestingly, Prontosan also resulted in a decrease between the nonpolarized and polarized Panex CE‐constructed e‐bandage treatment (*p* < 0.05), while Skintegrity did not. Lastly, when the Panex CE‐constructed e‐bandage was used with Purilon, there was a difference in bacterial burden between initial and polarized groups (*p* < 0.05), but not the nonpolarized and polarized groups, indicating that Purilon hydrogel may not be suitable for Panex CE‐constructed e‐bandage use (Figure 2d). Considering mean log_10_ (CFU/cm^2^) values of Panex CE‐constructed polarized (active) e‐bandages with the six different hydrogels after 24 h of treatment against MRSA IDRL‐6169, activity decreased in the following order: Xanthan gum (6.09 ± 1.24 log_10_[CFU/cm^2^]), 3M (6.15 ± 1.27 log_10_[CFU/cm^2^]), Solosite (6.6 ± 0.64 log_10_[CFU/cm^2^]), Prontosan (7.34 ± 0.28 log_10_[CFU/cm^2^]), Skintegrity (7.37 ± 1.03 log_10_[CFU/cm^2^]), Duoderm (7.95 ± 0.36 log_10_[CFU/cm^2^]) and Purilon (8.17 ± 0.26 log_10_[CFU/cm^2^]) (Figure 2).

For Elat CE‐constructed e‐bandages with Xanthan gum, there was a significant decrease in MRSA IDRL‐6169 between the nonpolarized and polarized e‐bandage groups (*p* < 0.05); there was also a difference between the initial and nonpolarized e‐bandage log_10_(CFU/cm^2^) (*p* < 0.05, Figure 3g). 3M, Prontosan, Purilon and Solosite hydrogels had similar activities regardless of the CE material (Figures 2a,c,d,f and 3a,c,d,f). Unlike Panex CE‐constructed e‐bandages (Figure 2b), Elat CE‐constructed e‐bandages with Duoderm hydrogel did not show statistically significant differences between the nonpolarized and polarized groups (Figure 3b). Elat CE‐constructed e‐bandages with Skintegrity hydrogel did not demonstrate significant bacterial reductions when comparing initial and polarized groups (Figure 3e). Regardless of CE material, there was no significant difference between nonpolarized and polarized e‐bandage groups when Skintegrity hydrogel was used against in vitro MRSA IDRL‐6169 biofilms. Considering mean log_10_(CFU/cm^2^) values of Elat CE‐constructed e‐bandages with the hydrogels after 24 h of treatment against MRSA IDRL‐6169, activity decreased in the following order: Solosite (6.39 ± 1.27 log_10_[CFU/cm^2^]), Duoderm (6.97 ± 0.80 log_10_[CFU/cm^2^]), Prontosan (7.36 ± 0.27 log_10_[CFU/cm^2^]), Skintegrity (7.40 ± 0.96 log_10_[CFU/cm^2^]), Xanthan gum (7.46 ± 0.28 log_10_[CFU/cm^2^]), 3M (7.70 ± 0.84 log_10_[CFU/cm^2^]) and Purilon (8.08 ± 0.19 log_10_[CFU/cm^2^]) (Figure 3).

### 3.2 Activity of Off‐the‐Shelf Hydrogels Against *A. baumannii* ATCC 17978 Biofilms

**FIGURE 4:**
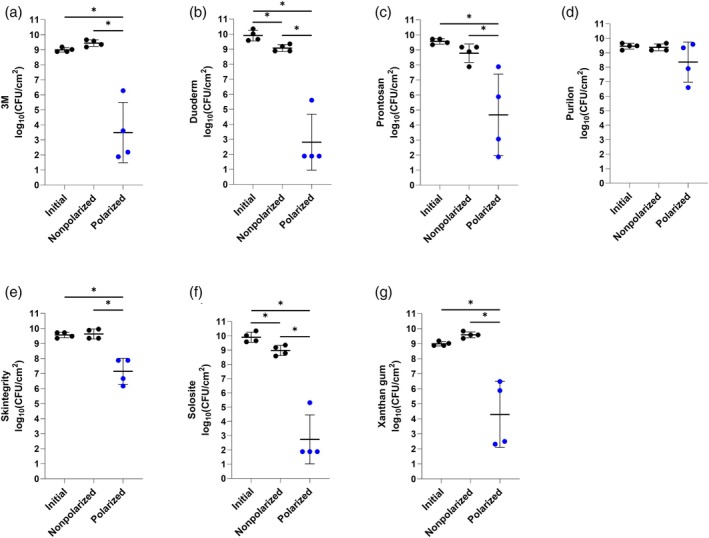
Results of testing of Panex counter and working electrode‐constructed e‐bandages against *Acinetobacter baumannii* ATCC‐17978 with (a) 3M, (b) Duoderm, (c) Prontosan, (d) Purilon, (e) Skintegrity, (f) Solosite and (g) Xanthan gum hydrogels. Polarized groups (shown in blue) had e‐bandages polarized for 24 h. Nonpolarized groups had e‐bandages applied but not polarized for 24 h. ‘Initial’ designates initial bacterial quantities (i.e., at time zero). Data points represent individual biological replicates (circles) and means (horizontal lines) with standard deviations of at least four independent biological replicates with statistically significant comparisons (*p < 0.05, two‐sided Wilcoxon rank‐sum test) shown.

**FIGURE 5:**
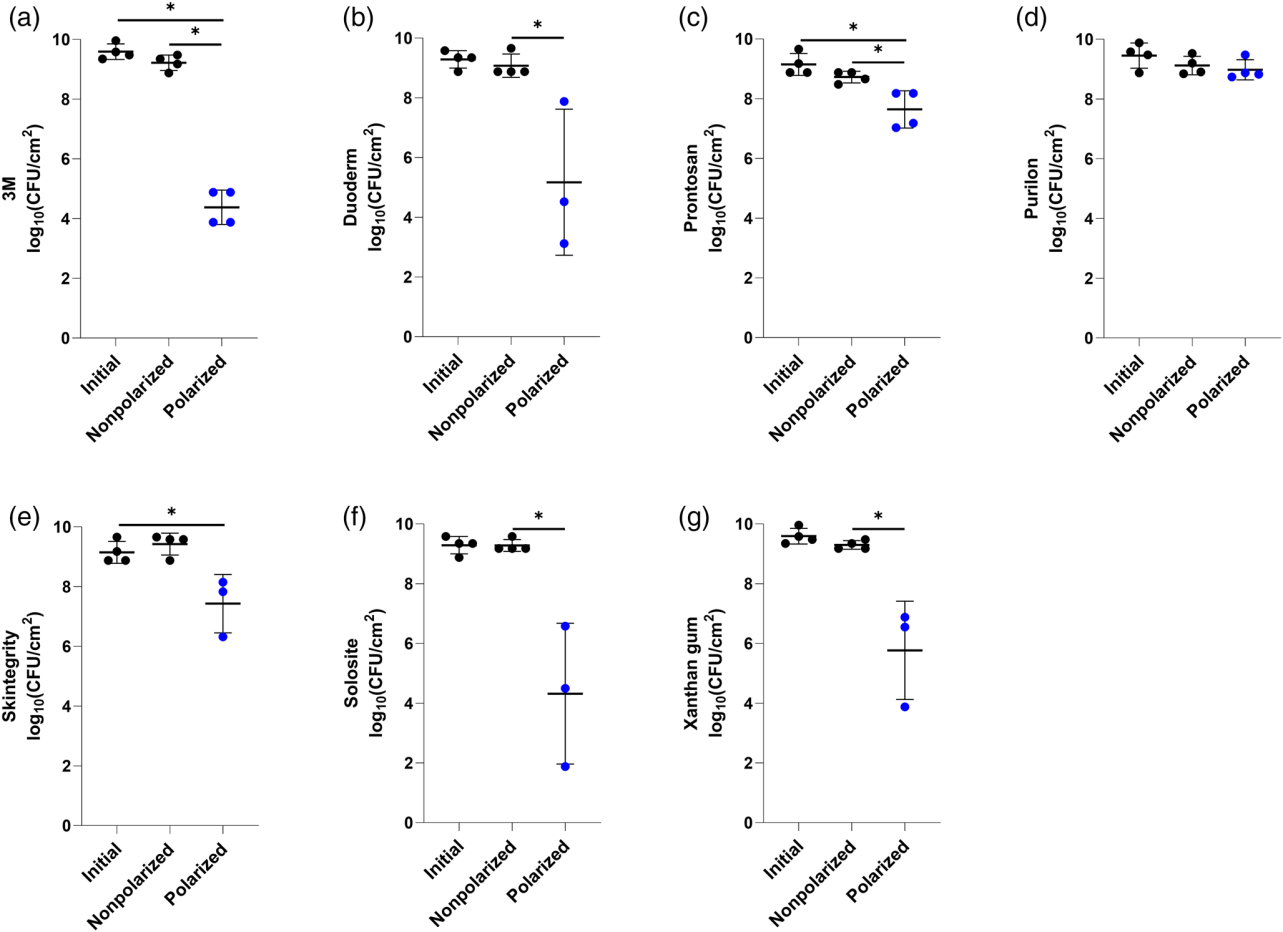
Results of testing of Elat counter electrode‐ and Panex working electrode‐constructed e‐bandages against *Acinetobacter baumannii* ATCC‐17978 with (a) 3M, (b) Duoderm, (c) Prontosan, (d) Purilon, (e) Skintegrity, (f) Solosite and (g) Xanthan gum hydrogels. Polarized groups (shown in blue) had e‐bandages polarized for 24 h. Nonpolarized groups had e‐bandages applied but not polarized for 24 h. ‘Initial’ designates initial bacterial quantities (i.e., at time zero). Data points represent individual biological replicates (circles) and means (horizontal lines) with standard deviations of at least four independent biological replicates with statistically significant comparisons (*p < 0.05, two‐sided Wilcoxon rank‐sum test) shown.

Figures 4 and 5 show e‐bandage activities with six off‐the‐shelf hydrogels and Xanthan gum against *A. baumannii* ATCC 17978 biofilms when using Panex or Elat carbon fabric as the CE, respectively.

Panex CE‐constructed e‐bandages using either Duoderm or Solosite hydrogel resulted in a decrease in microbial burden compared to the initial and nonpolarized groups (*p* < 0.05). There was also a microbial burden reduction between the initial and nonpolarized groups (*p* < 0.05, Figure 4b,f); when Elat CE‐constructed e‐bandages were combined with the same hydrogels, the only significant bacterial reduction was between the nonpolarized and polarized groups (Figure 5b,f). Panex CE‐constructed e‐bandages using 3M, Prontosan, Skintegrity or Xanthan gum hydrogel showed bacterial reductions in polarized groups compared to initial or nonpolarized groups (*p* < 0.05), while no significant reduction was observed between the initial and nonpolarized groups (Figure 4a,c,e,g). Additionally, when the control hydrogel, Xanthan gum, was polarized, a reduction in *A. baumannii* ATCC‐17978 biofilms (~5 log_10_[CFU/cm^2^]) was observed, consistent with previous findings [16]. Elat CE‐constructed e‐bandages using 3M or Prontosan did not change biocidal activity (Figure 5a,c) compared to Elat CE‐constructed e‐bandages. However, for Skintegrity and Xanthan gum hydrogels, changing the CE from Panex to Elat altered activity, with significant differences only found when comparing the polarized group to either the initial or nonpolarized groups (*p* < 0.05, Figure 5e,g). Finally, Purilon hydrogel‐operated e‐bandages did not show any significant differences between any group tested, regardless of CE material (Figures 4d and 5d), implying that Purilon hydrogel may not be suitable for e‐bandage use against *A. baumannii* ATCC‐17978.

Considering mean log_10_(CFU/cm^2^) values of Panex CE‐constructed e‐bandages with the six off‐the‐shelf hydrogels after 24 h of treatment against *A. baumannii* ATCC‐17978, activity decreased in the following order: Solosite (2.74 ± 1.72 log_10_[CFU/cm^2^]), Duoderm (2.81 ± 1.86 log_10_[CFU/cm^2^]), 3M (3.49 ± 2.01 log_10_[CFU/cm^2^]), Xanthan gum (4.29 ± 2.20 log_10_[CFU/cm^2^]), Prontosan (4.68 ± 2.72 log_10_[CFU/cm^2^]), Skintegrity (7.15 ± 0.87 log_10_[CFU/cm^2^]) and Purilon (8.36 ± 1.38 log_10_[CFU/cm^2^]) (Figure 4).

Considering mean log_10_(CFU/cm^2^) values of Elat CE‐constructed e‐bandages with the six hydrogels after 24 h of treatment against *A. baumannii* ATCC‐17978, activity decreased in the following order: Solosite (4.32 ± 2.36 log_10_[CFU/cm^2^]), 3M (4.38 ± 0.58 log_10_[CFU/cm^2^]), Duoderm (5.17 ± 2.45 log_10_[CFU/cm^2^]), Xanthan gum (5.77 ± 1.65 log_10_[CFU/cm^2^]), Skintegrity (7.43 ± 0.98 log_10_[CFU/cm^2^]), Prontosan (7.64 ± 0.62 log_10_[CFU/cm^2^]) and Purilon (8.98 ± 0.34 log_10_[CFU/cm^2^]) (Figure 5).

### 3.3 Increased Water Content of 3M Hydrogel Improves e‐Bandage Activity Against MRSA IDRL‐6169 Biofilms

**FIGURE 6:**
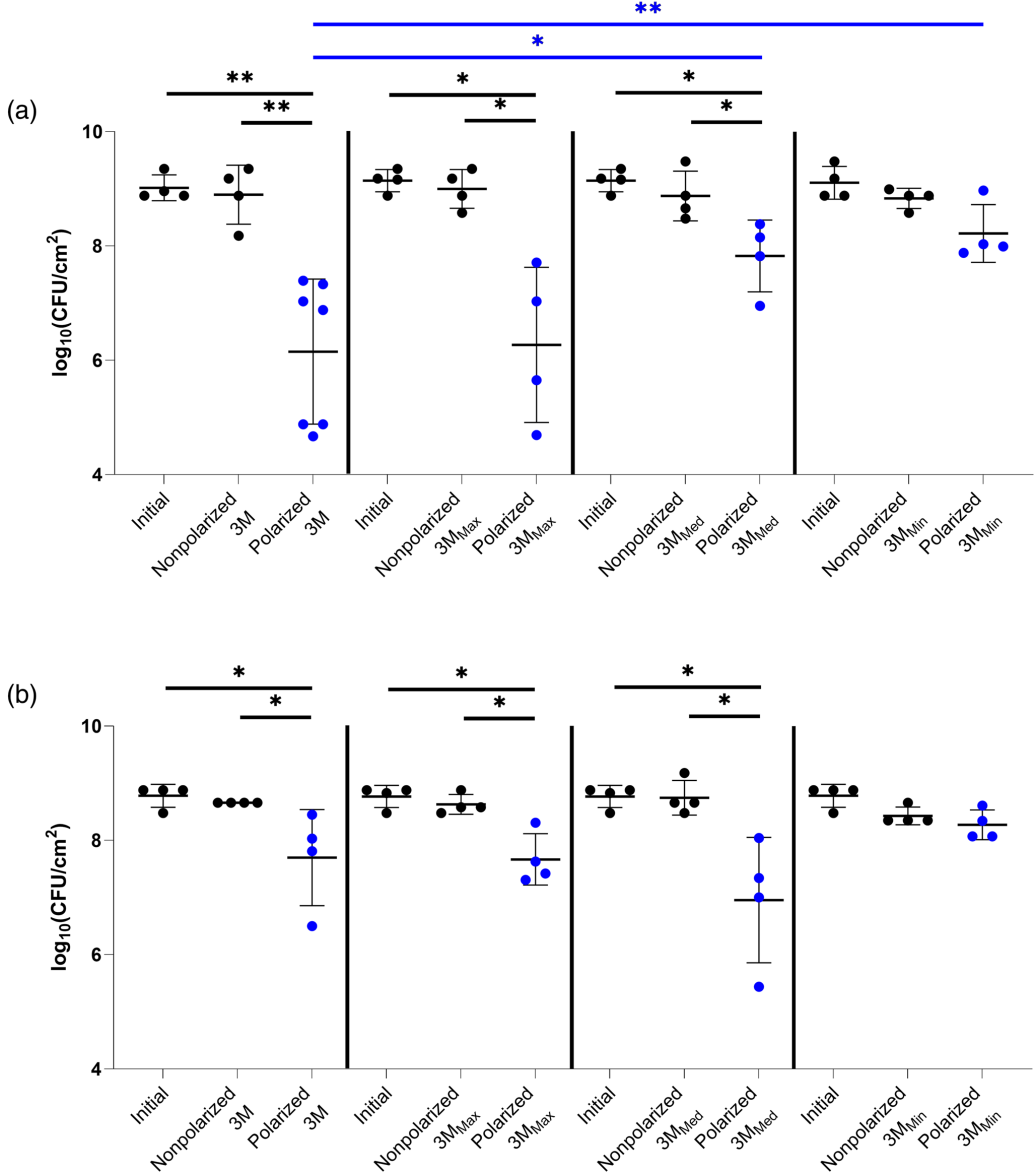
3M and 3M derived hydrogels (3M_Max_, 3M_Med_ and 3M_Min_) tested with e‐bandages against methicillin‐resistant *Staphylococcus aureus* IDRL‐6169. (a) Results with Panex CE‐constructed e‐bandages and (b) results with Elat CE‐constructed e‐bandages. Polarized groups (shown in blue) had e‐bandages polarized for 24 h. Nonpolarized groups had e‐bandages applied but not polarized for 24 h. ‘Initial’ designates initial bacterial quantities (i.e., at time zero). Data points represent individual biological replicates (circles) and their means (horizontal lines) with standard deviations of at least four independent biological replicates with statistically significant comparisons (*p < 0.05, **p < 0.01, two‐sided Wilcoxon rank‐sum test) shown. Blue statistical significance bars indicate cross hydrogel comparison for polarized groups.

3M hydrogel derivatives with water contents of 93% (3M_Max_), 84% (3M_Med_) and 74% (3M_Min_) were prepared [34]. Figure 6 shows activity of 3M hydrogel and its derivative hydrogels (3M_Max_, 3M_Med_ and 3M_Min_) against MRSA IDRL‐6169 biofilms with Panex or Elat CE‐constructed e‐bandages (Figure 6a,b, respectively). Off‐the‐shelf 3M, 3M_Max_ and 3M_Med_ hydrogels showed significant bacterial reductions between polarized and nonpolarized e‐bandages or initial groups (*p* < 0.01 and *p* < 0.05), regardless of the CE material used, with no differences between initial and nonpolarized groups. For 3M_Min_, there was no difference between the three groups. 3M_Med_ and 3M_Min_ resulted in lower e‐bandage activity compared to off‐the‐shelf 3M hydrogel (*p* < 0.05 and *p* < 0.01, respectively) using Panex but not Elat CE‐constructed e‐bandages. Overall, regardless of the CE material used, 3M_Max_ and 3M_Med_ derived hydrogels supported e‐bandage activity against MRSA IDRL‐6169.

### 3.4 Increased Water Content of 3M Hydrogel Improves e‐Bandage Activity Against *A. baumannii* ATCC 17978 Biofilms

**FIGURE 7:**
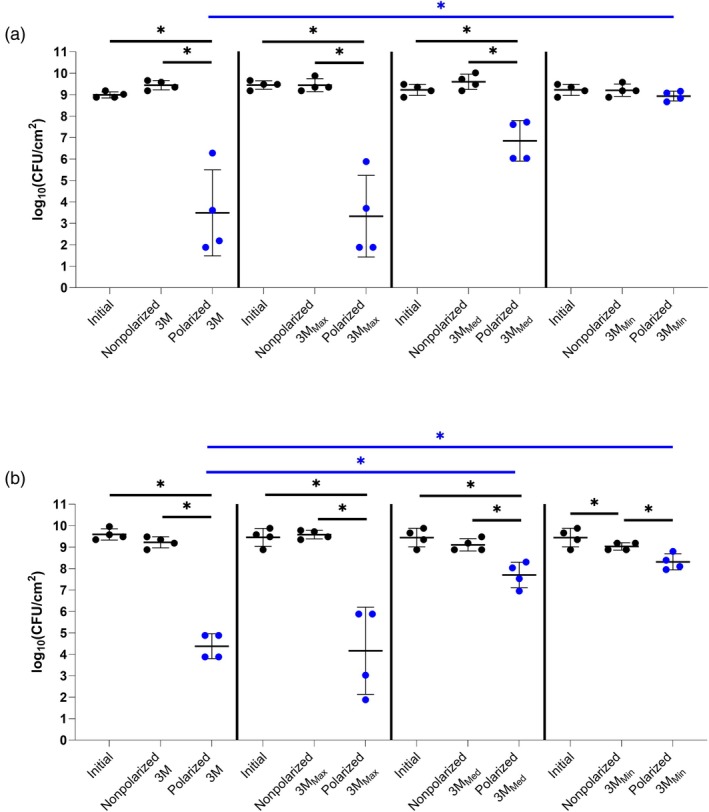
3M and 3M derived hydrogel (3M_Max_, 3M_Med_ and 3M_Min_) testing with e‐bandages against *Acinetobacter baumannii* ATCC‐17978 (a) shows results with Panex, and (b) Elat CE‐constructed e‐bandages. Polarized groups (shown in blue) had e‐bandages polarized for 24 h. Nonpolarized groups had e‐bandages applied but not polarized for 24 h. ‘Initial’ designates initial bacterial quantities (i.e., at time zero). Data points represent individual biological replicates (circles) and their means (horizontal lines) with standard deviations of at least four independent biological replicates with statistically significant comparisons (*p < 0.05, two‐sided Wilcoxon rank‐sum test) shown. Blue statistical significance bars indicate cross hydrogel comparison for polarized groups.

Figure 7 shows activities of 3M and 3M derived hydrogels against *A. baumannii* ATCC 17978 when the e‐bandage is used with Panex and Elat carbon fabric as the CE. Panex CE‐constructed e‐bandages with off‐the‐shelf 3M, 3M_Max_ or 3M_Med_ hydrogel all showed decreases in bacterial quantities when comparing polarized groups to initial or nonpolarized groups (*p* < 0.05), with no significant difference between initial and nonpolarized groups. 3M_Min_ hydrogel with Panex CE‐constructed e‐bandages showed no significant variance in bacterial burden when comparing polarized groups to initial or nonpolarized groups; however, Elat CE‐constructed e‐bandages demonstrated enhanced activity when comparing nonpolarized and polarized groups, with the only instance of variance in log_10_(CFU/cm^2^) being between the initial and nonpolarized groups (cause unknown). When comparing polarized groups with 3M_Max_, 3M_Med_ and 3M_Min_ hydrogels to those with 3M hydrogel, e‐bandages with 3M_Min_ were less active (*p* < 0.05) against *A. baumannii* ATCC‐17978 than those with off‐the‐shelf 3M hydrogel regardless of CE material. 3M_Med_ hydrogel also showed a reduced capacity to support e‐bandage activity when compared to off‐the‐shelf 3M hydrogel, but only with Elat CE‐constructed e‐bandages (*p* < 0.05). Regardless of the use of Panex or Elat as the CE, 3M_Max_ hydrogel performed at least as effectively as off‐the‐shelf 3M hydrogel against MRSA IDRL‐6169 and *A. baumannii* ATCC 17978 biofilms.

## 4 Discussion

Hydrogels are used in many applications in medicine [43]. They can provide biomechanical integration at tissue‐electrode interfaces [26]. The human body consists of a variety of soft, highly hydrated tissues, whereas e‐bandages are composed of carbon fibers and dry electrode components. Differences between the two surfaces necessitate the use of hydrogels to ensure effective electrochemical activity of an e‐bandage. A multitude of off‐the‐shelf hydrogels can potentially be utilized for individualized patient treatment. cPreviously, a prototype 1.77 cm^2^ e‐bandage was designed for wound care (in mice), functionality of which hinges on being coated in hydrogel. Considering the breadth of hydrogel products available, this study focused on functionality of the e‐bandage when operated with one of six off‐the‐shelf hydrogels and also evaluated the differential hydration of one hydrogel.

### 4.1 Off‐the‐Shelf Hydrogels in e‐Bandage Applications for Biocidal Activity

3M hydrogel is made of propylene glycol, guar gum and sodium tetraborate, and provides a moist healing environment for enhancing wound healing [34]. 3M hydrogel‐operated e‐bandages demonstrated a significant reduction in bacterial load against MRSA biofilms when either Panex (*p* < 0.01 vs. initial and nonpolarized) or Elat (*p* < 0.05 vs. initial and nonpolarized) was used as the CE. Moreover, with the two CE compositions, significant reductions were observed against *A. baumannii* biofilms (*p* < 0.05 vs. initial and nonpolarized).

Duoderm hydrogel contains a carboxymethylcellulose base [36] and is commonly used for wound healing, including as a positive control in many wound healing studies [44, 45, 46]. In this study, Duoderm hydrogel‐operated e‐bandages decreased MRSA and *A. baumannii* biofilms (*p* < 0.05 vs. initial or nonpolarized).

Prontosan hydrogel contains betaine and polyaminopropyl biguanide (polyhexadine, PHMB) [37]. Betaine is a surfactant which may modify bacterial surfaces resulting in biofilm and wound debris removal [47]. PHMB has been reported to have antimicrobial activity against Gram‐negative and Gram‐positive bacteria, as well as fungi [48]. In line with these reports, results of this study demonstrate that Prontosan hydrogel loaded nonpolarized e‐bandage shows antibacterial activity against MRSA IDRL‐6169 biofilms (*p* < 0.05 vs. initial and nonpolarized) (Figures 2 and 3). However, its activity against MRSA and *A. baumannii* biofilms increased when used to operate an H_2_O_2_‐producing e‐bandage, resulting in a statistically significant reduction in these bacteria compared to the initial condition and the nonpolarized groups (*p* < 0.05).

Purilon hydrogel consists of sodium carboxymethylcellulose and calcium alginate [38]. Alginate is used in biomedical science, especially in tissue engineering, including bone and soft tissues, due to its biocompatibility and gel formation properties [49, 50]. In the experiments conducted with Purilon hydrogel, e‐bandage activity against MRSA or *A. baumannii* biofilms was limited. Treatment resulted in a reduction of MRSA biofilms (*p* < 0.05 vs. initial) but showed no significant effect against *A. baumannii* , even when different CE materials were used. Electrochemical results indicated that Purilon hydrogel exhibited a distinct behavior compared to other hydrogels. It had the highest uncompensated resistance (Ru) amongst all tested hydrogels measured before bandage treatment (Table S1). Considering Ru values of the hydrogels after 24 h of H_2_O_2_ treatment, a substantial decrease was observed compared to initial values, indicating that the system reaches a stable equilibrium under polarization. This suggests that the observed reduction in resistance is associated with electrochemical activity induced by polarization. Additionally, unlike other tested hydrogels, the Faradaic current decreased over time; however, during Purilon hydrogel loaded H_2_O_2_ e‐bandage treatment (Figure S1).

Skintegrity hydrogel consists of allantoin, hydroxyethylcellulose, and dextran [39]. While allantoin provides moisture [51], hydroxyethylcellulose is a thickening, stabilizing and emulsifying agent [52], and dextran can enhance moisture retention, improve biocompatibility, and provide a scaffold for wound healing [53]. Therefore, this hydrogel may provide wound hydration and promote healing. Even when the e‐bandage used with Skintegrity hydrogel was not polarized, it showed antimicrobial activity against MRSA biofilms (*p* < 0.05 vs. initial). It also worked with H_2_O_2_‐producing e‐bandage. It had biocidal activity against MRSA (*p* < 0.05 vs. initial) when Panex, but not Elat, was used as the CE. *A. baumannii* biofilms were decreased significantly with the H_2_O_2_‐producing e‐bandage operated by Skintegrity hydrogel (*p* < 0.05) compared to the initial condition and the nonpolarized e‐bandages.

Solosite's active ingredients include methylparaben, propylparaben, allantoin and benzyl alcohol [40]. Parabens provide broad‐spectrum antimicrobial activity [54], while allantoin provides skin‐soothing, moisturizing, and healing properties [51]. Benzyl alcohol is used as a preservative in pharmaceuticals and cosmetics to impede microbial growth [55]. Solosite was shown to provide beneficial outcomes in the healing of experimentally induced burn wounds in rats [56]. In this study, Solosite was the most effective hydrogel when used with the H_2_O_2_‐producing e‐bandage, achieving a bacterial reduction of ~2 log_10_(CFU/cm^2^) for MRSA (*p* < 0.05) and ~6 log_10_(CFU/cm^2^) for *A. baumannii* (*p* < 0.05) compared to initial or nonpolarized groups.

Differences in antimicrobial activity observed amongst hydrogels applied with the polarized e‐bandage are assumed to be related to hydrogel compositions and, consequently, their electrochemical behavior under polarized conditions. While this could be explored by performing detailed chemical analyses of potential reaction products, and/or detailed analysis of deconstructed hydrogels, doing so was beyond the scope of the current work.

### 4.2 Evaluation of Customized Hydrogels for Enhanced e‐Bandage Activity

Xanthan gum and custom‐made 3M hydrogels were prepared and tested with e‐bandages. Xanthan gum has no independent antimicrobial activity [57], but exhibits excellent water solubility and biocompatibility, making it non‐toxic and non‐irritating to skin. It provides a beneficial moisturizing environment and supports wound healing [58]. As in previous work [15, 16, 17, 18, 19, 20, 59], Xanthan gum was compatible with the H_2_O_2_‐producing e‐bandages, showing activity against MRSA as well as *A. baumannii* biofilms when Panex (*p* < 0.01 vs. initial and nonpolarized) or Elat (*p* < 0.05 vs. initial and nonpolarized) was used as the CE.

Increasing hydrogel water content was assessed. 3M hydrogels were prepared with varying water content. This may allow enhanced moisturization of the wound environment during e‐bandage application, prolonged use of the same hydrogel, and potential cost reduction through large‐scale production. All 3M‐derived hydrogels worked with e‐bandages in at least one tested condition, although their biocidal activities varied. 3M_Max_ and 3M_Med_ showed a significant reduction in MRSA and *A. baumannii* loads compared to the initial and nonpolarized groups when applied with H_2_O_2_‐producing e‐bandages, whether Panex or Elat CEs were used (*p* < 0.05). However, 3M_Med_ (*p* < 0.05) and 3M_Min_ (*p* < 0.01) operated polarized e‐bandages exhibited lower biocidal activities than off‐the‐shelf 3M hydrogel with Panex CE‐operated e‐bandages against MRSA. On the other hand, when *A. baumannii* biofilms were tested, active e‐bandages with 3M_Min_ (*p* < 0.05, CE:Panex) and both 3M_Med_ and 3M_Min_ (*p* < 0.05, CE: Elat) hydrogels showed reduced biocidal activities compared to off‐the‐shelf 3M hydrogel‐operated e‐bandages. Overall, when applied with polarized e‐bandages, 3M_Max_ hydrogel, which provides higher water content, demonstrated antimicrobial activity at least equivalent to that of off‐the‐shelf 3M.

The effects of hydrogels alone, without the use of nonpolarized e‐bandages, were not assessed. Given that the hydrogels used are in direct contact with the biofilm, the presence of carbon fabric on top of the hydrogel is not expected to confer additional biocidal effects, as carbon fabrics are chemically inert.

## 5 Conclusions

H_2_O_2_‐producing e‐bandages showed biocidal activity against MRSA and *A. baumannii* biofilms when used with off‐the‐shelf or modified hydrogel, indicating their versatility. It is concluded that:

- All tested off‐the‐shelf hydrogels applied with polarized e‐bandages demonstrated comparable performance in at least one tested condition (initial/nonpolarized) to previously used Xanthan gum, except for Elat CE‐operated e‐bandages with Skintegrity hydrogel against MRSA and Elat‐ or Panex‐operated CE e‐bandages with Purilon hydrogel against *A. baumannii* .
- Increasing water content of 3M hydrogel to 93% (3M_Max_) provided antimicrobial e‐bandage activity at least equivalent to that of off‐the‐shelf 3M against both MRSA and *A. baumannii* biofilms.
  Pairing individual hydrogels with individual electrode materials (Elat or Panex carbon fabric) may impact e‐bandage activity, although some hydrogels (e.g., 3M) performed well regardless of CE material or bacterium studied.

## Conflicts of Interest

H.B. holds a patent (US20180207301A1), ‘Electrochemical reduction or prevention of infections’ which refers to the electrochemical bandage described herein. The other authors declare no conflicts of interest.

## Supporting information


**Figure S1:** The current–time graphics of the hydrogels with e‐bandages on *Acinetobacter baumannii* during 24 h H_2_O_2_ treatment (CE: Elat, WE: Panex). Custom made 3M hydrogels containing 3M_Max_: 93% water, 3M_Med_: 84% water and 3M_Min_: 74% water.
**Table S1:** Hydrogels' uncompensated resistance (Ru) values before and after 24 h of H_2_O_2_ treatment.

## Data Availability

The data that support the ﬁndings of this study are available from the corresponding author upon reasonable request.
