# Supplementary material for: H2O2 ‐Producing Electrochemical Bandages Are Active Using Off‐the‐Shelf Hydrogels
Source: Wound Repair Regen. 2025 Sep 15;33(5):e70092. doi: 10.1111/wrr.70092 (PMC12438947; doi:10.1111/wrr.70092)
Supplement: Supplementary file 1 — Figure S1: The current–time graphics of the hydrogels with e‐bandages on Acinetobacter baumannii during 24 h H2O2 treatment (CE: Elat, WE: Panex). Custom made 3M hydrogels containing 3MMax: 93% water, 3MMed: 84% water and 3MMin: 74% water. Table S1: Hydrogels' uncompensated resistance (Ru) values before and after 24 h of H2O2 treatment. [file WRR-33-0-s001.docx]

Supporting Information

**H2O2-producing electrochemical bandages are active using off-the-shelf hydrogels**

Eda Dagsuyu^a,b^, Paige Kies^c^, Robin Patel^c,d^, Haluk Beyenal^a*^

^a^School of Chemical Engineering and Bioengineering, Voiland College of Engineering and Architecture, Washington State University, Pullman 99164, Washington, United States of America

^b^Department of Chemistry, Faculty of Engineering, Istanbul University-Cerrahpaşa, Istanbul 34320, Türkiye

^c^Division of Clinical Microbiology, Mayo Clinic, Rochester 55905, Minnesota, United States of America

^d^Division of Public Health, Infectious Diseases, and Occupational Medicine, Department of Medicine, Mayo Clinic, Rochester 55905, Minnesota, United States of America

**Electrochemical Activity Studies**

Most hydrogels had no electrochemically adverse effects on e-bandage operation (Figure S1). Measured internal resistances are shown in Table S1. The electrochemical response when different hydrogels were employed varied without demonstrating significant correlation to biocidal activity. The current-time graphics of 3M_Med_, 3M_Min_ and Purilon hydrogels have decreasing trends (current-time) which are also shown (purple color) in Figure S1; also, their uncompensated resistance (Ru) values are higher than other hydrogels (Table S1) and their biocidal activities are lower than other hydrogels. Ideally, the lower the Ru, the better the electrochemical. However, this may not be directly translated to H_2_O_2_ production and diffusion in the hydrogel. In addition, changes in Ru values before and after use for 3M_Min_, Duoderm, Prontosan, Purilon, Solosite, Skintegrity, and Xanthan gum hydrogels were noted (*p*<0.05).


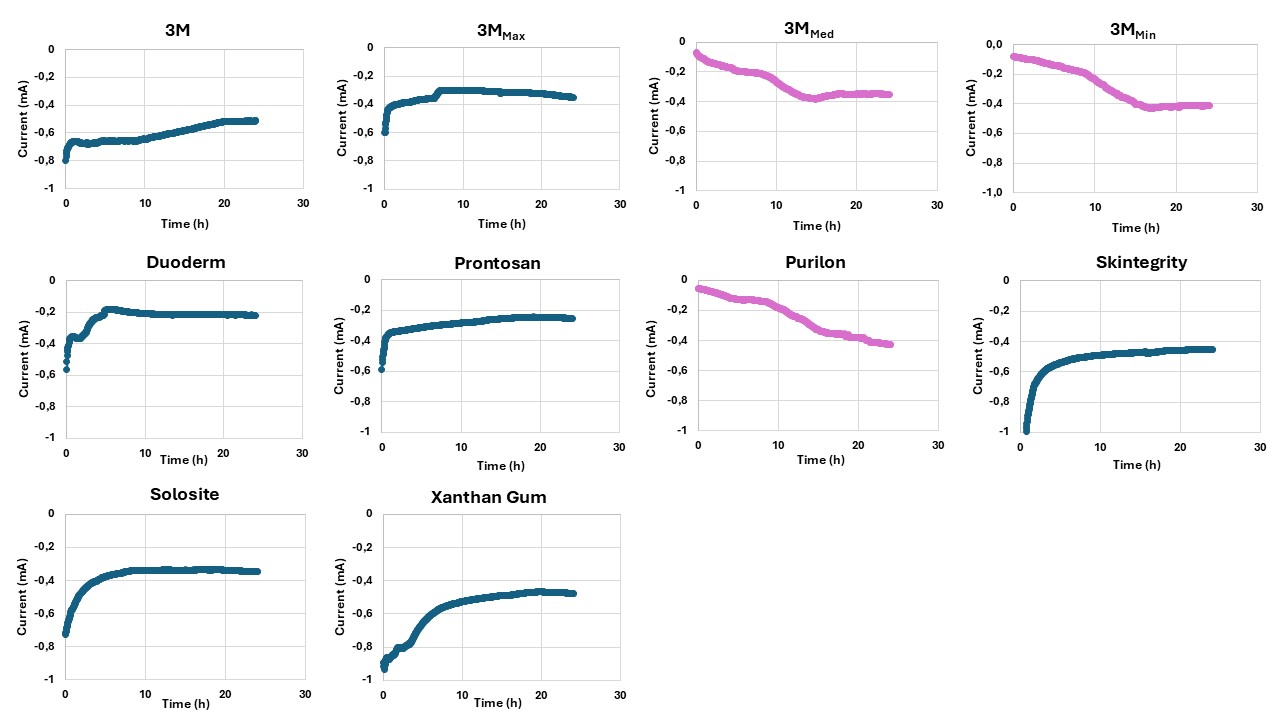


**Figure S1.** The current-time graphics of the hydrogels with e-bandages on A. baumannii during 24 hours H_2_O_2_ treatment (CE: Elat, WE: Panex). Custom made 3M hydrogels containing 3M_Max_: 93% water, 3M_Med_: 84% water and 3M_Min_: 74% water.

**Table S1.** Hydrogels' uncompensated resistance (Ru) values before and after 24 hours of H_2_O_2_ treatment.

| Hydrogels | Ru_Before_, Ohm* | Ru_After_, Ohm* |
| --- | --- | --- |
| 3M | 73±43 | 22±6 |
| 3M_Max_ | 27±9 | 15±8 |
| 3M_Med_ | 248±61 | 112±38 |
| 3M_Min_ | 251±89 | 57±29 ^a^ |
| Duoderm | 76±17 | 16±4 ^a^ |
| Prontosan | 21±3 | 12±1 ^a^ |
| Purilon | 295±222 | 43±5 ^a^ |
| Solosite | 30±11 | 15±2 ^a^ |
| Skintegrity | 25±9 | 11±1 ^a^ |
| Xhantan Gum | 29±10 | 11±4 ^a^ |

*Data are presented as the mean of four replicates ± standard deviation with statistically significant comparisons (^a^*p*<0.05 vs. Ru_Before_, two-sided Wilcoxon rank-sum test).
